# Hospital nurse staffing and staff–patient interactions: an observational study

**DOI:** 10.1136/bmjqs-2018-008948

**Published:** 2019-03-27

**Authors:** Jackie Bridges, Peter Griffiths, Emily Oliver, Ruth M Pickering

**Affiliations:** 1 School of Health Sciences, University of Southampton, Southampton, UK; 2 NIHR Collaboration for Leadership in Applied Health Research and Care for Wessex, Southampton, UK; 3 Primary Care and Population Sciences, University of Southampton, Southampton, UK

**Keywords:** health services research, nurses, patient-centred care

## Abstract

**Background:**

Existing evidence indicates that reducing nurse staffing and/or skill mix adversely affects care quality. Nursing shortages may lead managers to dilute nursing team skill mix, substituting assistant personnel for registered nurses (RNs). However, no previous studies have described the relationship between nurse staffing and staff–patient interactions.

**Setting:**

Six wards at two English National Health Service hospitals.

**Methods:**

We observed 238 hours of care (n=270 patients). Staff–patient interactions were rated using the Quality of Interactions Schedule. RN, healthcare assistant (HCA) and patient numbers were used to calculate patient-to-staff ratios. Multilevel regression models explored the association between staffing levels, skill mix and the chance of an interaction being rated as ‘negative’ quality, rate at which patients experienced interactions and total amount of time patients spent interacting with staff per observed hour.

**Results:**

10% of the 3076 observed interactions were rated as negative. The odds of a negative interaction increased significantly as the number of patients per RN increased (p=0.035, OR of 2.82 for ≥8 patients/RN compared with >6 to <8 patients/RN). A similar pattern was observed for HCA staffing but the relationship was not significant (p=0.056). When RN staffing was low, the odds of a negative interaction increased with higher HCA staffing. Rate of interactions per patient hour, but not total amount of interaction time, was related to RN and HCA staffing levels.

**Conclusion:**

Low RN staffing levels are associated with changes in quality and quantity of staff–patient interactions. When RN staffing is low, increases in assistant staff levels are not associated with improved quality of staff–patient interactions. Beneficial effects from adding assistant staff are likely to be dependent on having sufficient RNs to supervise, limiting the scope for substitution.

## Background

Controlling health system expenditure has been an important policy goal in the UK National Health Service (NHS) and many similar publicly funded health systems.^1–3^ The tension between the need for greater efficiency and the impact on quality and safety has been compounded by shortages of registered nurses (RNs). In the UK, these shortages have been created by reduced intakes to RN training coupled with severe RN recruitment and retention difficulties.^4 5^ Service managers have been driven to consider reducing RN staffing levels and diluting the skill mix of nursing teams, substituting RN posts with healthcare assistants (HCAs) who are unregistered and for whom no formal training is required.^6–9^ Wide variations in staffing policies and practices persist across the NHS, and there is a lack of consensus in many countries on the value of mandated staffing levels and appropriate nursing skill mix.^10–12^ In the NHS, a number of concerns have been raised about the ability of HCAs to deliver complex compassionate care, although at the same time the relational abilities of RNs have also been widely questioned.^13–16^

Many studies report a relationship between low nurse staffing levels and adverse outcomes, particularly higher mortality rates. These findings are reported in studies across the globe including the UK and wider Europe, Australia, China, Thailand and USA.^6^ A smaller number of studies have reported associations between low staffing levels and low quality of care or patient satisfaction.^17–19^ The National Institute for Health and Social Care Excellence (NICE), the body which provides guidance and sets standards of care for the NHS called for more evidence considering skill mix and outcomes related to patient satisfaction to guide policy and practice on hospital nurse staffing.^6 9^

Research on nurse staffing levels and skill mix, while international and large scale, has relied on retrospective methods and rarely examines the staffing experienced by individual patients, which hampers the weight of subsequent staffing recommendations and thus impact on policy and practice. Most studies use hospital administrative data to gather staffing information, linking staffing to patients as a hospital average over a period of time, which does not reflect the care received by an individual on a particular ward on a specific day.^6 7^

Qualitative studies of RN views report that a lack of nursing time interferes with RNs building relationships with patients in hospital.^20^ However, the extent to which hospital nurse staffing levels or skill mix actually impact on the quality of interaction with patients has not been reported before. Quality of interaction between staff and hospital patients is an important determinant of patient experience and wider quality of care, especially for older patients with complex needs related to dementia or communication impairments.^20–24^ The study reported here aimed to explore association between nurse staffing levels, skill mix and the quality and quantity of daytime interactions with patients in hospital wards.

## Methods

This is a secondary analysis of observational data collected as part of a feasibility study of a compassionate care intervention for hospital nursing teams.^25^ The study took place in two NHS hospital Trusts in England that collectively employ an estimated 13 800 staff members. Six wards, across the two sites, with high proportions of patients aged over 65 participated: medicine for older people (four wards), urology (one ward) and orthopaedics (one ward). Each study ward had between 28 and 32 beds and almost 100% bed occupancy, with an average of 44 full-time equivalent nursing staff (RNs and HCAs) employed.

We used the Quality of Interaction Schedule (QuIS),^26^ an observational, time sampling tool that gives a measure of the length and quality of interactions between staff and patients on the ward. It has been used in a number of studies in NHS acute care settings for service improvement and evaluation.^27–30^ Interactions are rated as positive social, positive care, neutral, negative protective or negative restrictive. In this paper, we focus on interactions classified as ‘negative’ (negative protective or negative restrictive), an approach taken by other researchers using QuIS.^29^ In feasibility testing, we found close agreement between pairs of observers in relation to the number of interactions observed and moderate to substantial agreement on the QuIS rating.^31 32^ We also found reasonable correlation between QuIS and patients’ ratings.^31^

Observations took place between March 2015 and March 2016 and were undertaken by one of 12 trained observers during each of 120 2-hour sessions. Timing of sessions was balanced between wards, day of week and time of day (Monday–Friday, 08:00–22:00). Within each observation session, an index patient was chosen at random from all eligible patients on the ward in question and invited to take part in the study. Patients were excluded if they were unable to communicate their choices about taking part in the research and a consultee (as defined by the Mental Capacity Act 2005 Code of Practice)^33^ could not be consulted. Patients who indicated either verbally or non-verbally that they did not wish to take part were excluded, as were patients who were unconscious or those for whom there were clinical concerns that precluded them from being approached. Patients excluded for clinical reasons included people who were critically ill, at the end of life or isolated because of a high risk of infection. If the index patient approached consented to take part, up to three other patients in their vicinity were also approached and invited. If the patient declined to take part, a new index patient was selected. This process continued until an index patient was consented.

We developed a protocol to guide the observers in making their QuIS ratings.^31^ Characteristics of the selected patients (gender, age, cognitive impairment) were recorded, along with session characteristics (number of patients, RNs and HCAs on the ward at the start of the session). During the 2-hour observation session, the observer would find a discrete location on the ward where they were able to observe the social interactions between staff of any discipline and the index and other recruited patients. Observations were recorded in real time using tablet-based software (QI Tool).^25^ For each interaction, the following data were recorded: its start and finish time; the quality of the interaction; staff type and number of staff involved; whether or not the patient was agitated and whether or not visitors were present.

For analysis purposes, RN staffing was categorised as low (eight or more patients per RN, reflecting the threshold for low NHS staffing recognised by NICE),^9^ medium (6.1–7.9 patients per RN) and high (six or fewer patients per RN).^9^ Because there is no equivalent guidance defining low HCA staffing, we divided HCA staffing levels into tertiles based on observation sessions reflecting low (over eight patients per HCA), medium (7–8 per HCA) and high staffing (fewer than seven patients per HCA). Descriptive statistics (means and SD) for patient, observation session and interaction characteristics were calculated.

A three-level mixed logistic regression model was estimated using the command xtmelogit in Stata V.11.0,^34^ to investigate the impact of staffing levels on the chance of an interaction being rated negatively: the lowest level being the individual interaction and the higher two levels represented by random effects for the patient and observation session involved. The three-category variables for RN and HCA staffing level were included as fixed factors along with the following controlling variables: patient age, gender and cognitive impairment; presence of visitors, patient agitation and staff type and the ward involved. We explored the impact of each fixed effect alone (including only patient and session-level random effects in the variance structure) and in the presence of all others. Finally, because the influence of one staff group may be related to that of another, specific combinations of RN and HCA staffing were examined by including an interaction term in the model. Analysis was repeated for the subset of interactions involving at least one member of nursing staff (ie, an RN, an HCA, a student nurse or nursing staff not specified).

Association with a negative QuIS rating is presented as an odds ratio (OR) or adjusted OR (aOR) with 95% CIs. The significance of staffing level variables was tested postestimation using Wald tests at the 5% two-sided level. The Wald test for interaction (the need for combinations of RN and HCA staffing to be included in the model) was done in the presence of RN and HCA staffing main effects. The significance of other variables can be judged from the exclusion of the value 1.0 (representing equality) from the 95% CI around an OR/aOR.

The rate at which patients experienced interactions with staff per observed hour was explored in a negative binomial model at the patient level using command nbreg in Stata. Staffing level variables (and their combination) were examined on their own and after controlling for patient characteristics and the ward involved. Association is estimated from the model as incidence rate ratios (IRR) or adjusted IRRs along with 95% CIs. Statistical significance was judged as described above. The number of interactions experienced by each patient was the dependent variable in the negative binomial model, and the logarithm of their observed time was included as an offset. The median and IQR of the rate (number of interactions per observed hour) across patients are presented.

The amount of time a patient spent interacting with staff per observed hour was examined in normal-based models using command mixed in Stata, including observation session as a random effect, and the staffing level variables, patient characteristics and ward as fixed effects. Models were also examined for the logarithm of the percentage of time spent interacting per observed hour and estimates were back transformed to yield multiplicative effects.

Patients gave informed consent before taking part. The study took an inclusive approach to people with cognitive impairment, in order to properly reflect the relevant patient population. Procedures used were informed by requirements of the Mental Capacity Act and process consent.^33 35^ Process consent assumes residual capacity exists and then uses knowledge about how the person makes and communicates their choices and preferences in everyday situations as a basis for negotiating participation or not in the research. As required by the Mental Capacity Act, where capacity to decide to participate could not be established, a personal consultee was consulted.

## Results

Data were analysed from 119 2-hour sessions in which the care of 270 patients was observed (one session was omitted as staffing data were not recorded). Patients were aged between 18 and 101 years, with a mean of 82 years. Most patients were female (77%, n=209), and 25% (n=68) had evidence of cognitive impairment.

Across the 119 observation sessions on average 4.5 RNs (SD 1.2, range 2–8) and 4.1 HCAs (SD 1.2, range 0–9) were present on the ward at the observation session start, including bank and agency staff. There was a mean of 6.9 patients per RN (SD 1.7, range 4–13) and 8.5 patients per HCA (SD 2.4, range 3–18).

A total of 3076 interactions between patients and staff were observed, of which 299 (10%) were QuIS rated as negative. In the multivariate model, the odds of a negative rating were significantly higher with increasing patient age and patient agitation and when more than one member of staff was involved in the interaction. The odds were significantly lower when visitors were present (table 1).

**Table 1:** Unadjusted OR and aOR of a negative interaction among all QuIS rated interactions (n=3076)

Characteristic (number, % with characteristic)	Univariate* OR (95% CI)	Adjusted for all predictors* aOR (95% CI) (n=3076)
**Patient** **characteristics**		
Age (per unit increase)	1.03 (1.01 to 1.06)	1.03 (1.00 to 1.06)
Male (n=688, 22%)	1.10 (0.49 to 2.47)	1.56 (0.63 to 3.88)
With dementia (n=801, 26%)	1.32 (0.79 to 2.22)	1.07 (0.63 to 1.83)
**Interaction characteristics**		
Visitors present (n=191, 6%)	0.11 (0.04 to 0.33)	0.13 (0.04 to 0.41)
Patient agitated (n=92, 3%)	6.70 (3.34 to 13.43)	6.08 (2.97 to 12.48)
Staff type		
Registered nurse (n=925, 30%)	1.00	1.00
Student nurse (n=52. 2%)	0.98 (0.29 to 3.34)	1.07 (0.31 to 3.69)
HCA (n=1,017, 33%)	0.95 (0.66 to 1.37)	0.95 (0.66 to 1.38)
Doctor (n=88, 3%)	1.10 (0.47 to 2.55)	1.21 (0.51 to 2.89)
Allied HP (n=76, 2%)	1.17 (0.51 to 2.67)	1.28 (0.55 to 2.95)
Nurse/HCA not specified (n=618, 20%)	0.88 (0.58 to 1.34)	0.94 (0.61 to 1.44)
More than 1 member of staff (n=300, 8%)	2.30 (1.45 to 3.65)	2.38 (1.48 to 3.83)
**Observation session characteristics**		
Number of patients per RN	(P=0.097)	(P=0.035)
≤6 (n=1120, 36%)	0.97 (0.44 to 2.14)	0.90 (0.32 to 2.47)
>6 to <8 (n=1218, 40%)	1.00	1.00
≥8 (n=740, 24%)	2.32 (0.98 to 5.51)	2.82 (1.10 to 7.22)
Number of patients per HCA	(P=0.145)	(P=0.056)
<7 (n=1108, 36%)	0.50 (0.22 to 1.15)	0.38 (0.14 to 0.99)
7 to 8 (n=1193, 39%)	1.00	1.00
>8 (n=775, 25%)	1.10 (0.49 to 2.47)	1.24 (0.50 to 3.10)
**Ward**		
A (reference)	1.00	1.00
B	0.58 (0.19 to 1.75)	0.49 (0.12 to 1.93)
C	1.50 (0.50 to 4.47)	0.85 (0.23 to 3.18)
D	0.75 (0.24 to 2.32)	0.93 (0.27 to 3.27)
E	0.61 (0.20 to 1.88)	0.61 (0.15 to 2.38)
F	0.51 (0.15 to 1.73)	0.56 (0.15 to 2.03)

*All models include observation session and patient as random effects.

HCA, healthcare assistant; HP, health practitioner; QuIS, Quality of Interaction Schedule; RN, registered nurse; aOR, adjusted OR.

The odds of a negative interaction were not significantly associated with the staff group involved, although 94% of all interactions were with nurses or HCAs; the numbers of interactions for other staff groups were low. RN staffing levels were related to the overall rate of negative interactions with all staff members (p=0.035). When each RN was looking after high numbers (≥8 patients), the (adjusted) odds of a negative interaction were increased nearly threefold (aOR=2.82, 95% CI 1.10 to 7.22, in comparison to the reference staffing category of >6 to<8 patients per RN). HCA staffing levels were not significantly associated with the rate of negative interactions (p=0.056), although the pattern was similar to that for RN staffing. (table 1).

Focusing on the 2879 interactions involving a member of nursing staff (table 2), results show a trend of lower risk of a negative interaction with fewer patients per staff member and higher risk with more patients per staff member compared with the central category of staffing, for both RN (p=0.021) and HCA staffing (p=0.035). The model was significantly improved (p=0.024) when combinations of RN and HCA staffing were examined specifically. When RN staffing was high (≤6 patients per RN) or medium (>6 to <8), there were low rates of negative interactions when HCA staffing was high (<7 patients per HCA); but when RN staffing was low (≥8 patients per RN), there was a high rate of negative interactions when HCA staffing was high (table 2).

**Table 2:** Number (%) of negative QuIS ratings out of staff/patient interactions involving an RN, an HCA, a student nurse or unspecified RN/HCA, by RN and HCA staffing level, along with aORs (95% CI) of a negative QuIS rating in comparison to the reference combination (n=2879)

Number (%), negative QuIS OR (95% CI)		Patients per HCA			Split by RN staffing ratio
		<7	7–8	>8	
**Patients per RN**	**≤** **6**	11/373 (3%) 0.16 (0.03 to 0.73)	23/242 (10%) 0.81 (0.18 to 3.70)	48/414 (12%) 1.50 (0.35 to 6.42)	82/1029 (8%) 0.89 (0.31 to 2.55)
	**>6 to <** **8**	4/256 (2%) 0.09 (0.02 to 0.50)	70/656 (11%) 1.00	22/236 (9%) 0.87 (0.23 to 3.26)	96/1148 (8%) 1.00
	**≥** **8**	63/418 (15%) 2.12 (0.62 to 7.30)	24/217 (11%) 0.85 (0.22 to 3.29)	11/67 (16%) 1.05 (0.18 to 6.21)	98/702 (14%) 3.16 (1.20 to 8.36)
**Split by HCA staffing ratio**		78/1047 (7%) 0.34 (0.13 to 0.94)	117/1115 (11%) 1.00	81/719 (11%) 1.36 (0.53 to 3.49)	**Whole group** 276/2879 (10%)

*Controlled for patient, interaction and ward characteristics, with observation session and patient included as random effects. Main RN staffing level effect, p=0.021; main HCA staffing level effect, p=0.035.

HCA, healthcare assistant; QuIS, Quality of Interaction Schedule; RN, registered nurse.

On average, patients experienced 5.8 (median 5.5; IQR 3.5–7.5; range 0.5–20.5) interactions (with any staff member including non-nursing) per observed hour, many of which were less than a minute in length (mean 98 s; median 35 s). Only the staffing level variables were predictors of the rate at which patients experienced interactions (table 3), and though statistically significant (p=0.035), no clear trend emerged with RN staffing (both high and low staffing level categories having moderately increased adjusted IRRs (rates of interaction)). When HCAs were caring for high numbers (>8 patients), the rate of interactions was reduced (aOR=0.75, 95% CI 0.64 to 0.88, p=0.001 for difference among the three categories). The model was not significantly improved (p=0.062) when RN and HCA staffing combinations were additionally included. Neither staffing variable made a significant impact on the percentage of observed time patients spent interacting with staff: on average, patients had 9.5 (median 7.5; IQR 4.1–13.0; range 0.0–50.6) min of interaction time per observed hour. Results from the model for the logarithm of percentage time are shown in table 4. A similar picture of no significant predictors emerged from the model of unlogged percentage time (not shown).

**Table 3:** Unadjusted and adjusted IRR of an interaction with any member of staff

Characteristic	Univariate IRR (95% CI)	Adjusted for all predictors* Adjusted IRR (95% CI)
**Patient characteristics**		
Age (per unit increase)	1.01 (1.00 to 1.01)	1.00 (1.00 to 1.01)
Male	0.99 (0.85 to 1.14)	0.98 (0.84 to 1.14)
With dementia	1.02 (0.89 to 1.17)	1.01 (0.88 to 1.16)
**Observation session characteristics**		
Number of patients per RN	(p=0.331)	(p=0.035)
≤6	1.03 (0.89, 1.18)	1.20 (1.01, 1.43)
>6 to <8 (reference)	1.00	1.00
≥8	1.13 (0.96, 1.32)	1.21 (1.03, 1.43)
Number of patients per HCA	(p=0.000)	(p=0.001)
<7	1.02 (0.89, 1.18)	0.97 (0.83, 1.13)
7 to 8 (reference)	1.00	1.00
>8	0.76 (0.65, 0.88)	0.75 (0.64, 0.88)
Ward		
A (reference)	1.00	1.00
B	1.31 (1.08 to 1.59)	1.37 (1.08 to 1.72)
C	0.86 (0.70 to 1.05)	0.86 (0.67 to 1.09)
D	1.12 (0.91 to 1.37)	1.07 (0.86 to 1.34)
E	0.93 (0.76 to 1.14)	0.89 (0.71 to 1.13
F	0.84 (0.68 to 1.03)	0.93 (0.75 to 1.15)

Patient level analysis (n=270).

*Observation session included as a random effect.

HCA, healthcare assistant; IRR, incidence rate ratios; RN, registered nurse.

**Table 4:** Adjusted normal-based models for logged percentage time spent interacting with staff

Characteristic	Univariate* Multiplicative effect (95% CI)	Adjusted for all predictors Multiplicative effects (95% CI)
**Patient characteristics**		
Age (per unit increase)	1.01 (1.00 to 1.02)	1.00 (0.99 to 1.02)
Male contrasted to females	0.96 (0.67 to 1.38)	0.99 (0.67 to 1.46)
With (vs without) dementia	1.08 (0.82 to 1.44)	1.10 (0.82 to 1.48)
**Observation session characteristics**		
Number of patients per RN	(p=0.704)	(p=0.300)
≤6	1.12 (0.79, 1.60)	1.36 (0.88, 2.09)
>6 to <8 (reference)	1.00	1.00
≥8	1.16 (0.78, 1.74)	1.30 (0.85, 1.99)
Number of patients per HCA	(p=0.045)	(p=0.130)
≤7	0.81 (0.57, 1.17)	0.77 (0.51, 1.17)
7 to 8 (reference)	1.00	1.00
>8.00	0.63 (0.44, 0.91)	0.66 (0.44, 1.00)
Ward		
A (reference)	1.00	1.00
B	1.09 (0.66 to 1.81)	1.15 (0.62 to 2.12)
C	0.65 (0.39 to 1.08)	0.62 (0.34 to 1.16)
D	0.70 (0.42 to 1.19)	0.72 (0.41 to 1.26)
E	0.80 (0.48 to 1.32)	0.78 (0.42 to 1.43)
F	0.55 (0.33 to 0.93)	0.64 (0.37 to 1.11)

Patient level analysis (n=270).

*Observation session included as a random effect.

HCA, healthcare assistant; RN, registered nurse.

## Discussion

This is the first study to directly observe the association between nurse staffing and the quality and quantity of interactions with patients. Most interactions were rated as positive, but 10% attracted negative ratings. Staff type was not associated with the odds of a negative interaction. However, we did find that when RNs were caring for more patients, the odds of a negative interaction with any staff type were increased. Association between HCA staffing and quality of interactions depended on the level of RN staffing. While high HCA staffing was associated with reduced rates of negative interactions when in combination with medium or high RN staffing, when RN staffing was low and HCA staffing was high the rate of negative interactions was, if anything, increased. We found that the rate at which patients experienced interactions increased with high and low number of patients per RN and decreased with high number of patients per HCA. The amount of time patients spent in interactions was not significantly associated with staffing levels for either HCAs or RNs.

These findings are consistent with those from other studies which show that higher patient-to-staff ratios are associated with lower quality of care and patient satisfaction,^17 18^ but they shine an important and novel light on the impact on relational care, which may explain an important mechanism leading to poor patient experience. When there were eight or more patients per RN, the odds of a negative interaction more than doubled. Our findings could arise if nurses’ work patterns adapt to short staffing with an increased interaction rate, but one which has negative consequences. These findings are consistent with nurses’ views, reported in qualitative studies, that adequate staffing levels are an important antecedent to good relational care.^20 36^

Our study is the first to explore the role of assistant staff in the quality of interactions experienced. Whereas most previous research has reported negative associations between increased assistant staffing or diluted skill mix and outcomes, our findings provide a more nuanced picture that points to the important role that assistant staff play in maintaining the quality of care. While some accounts suggest that reported deficiencies in relational care provided by nurses are the result of interactions with poorly trained assistants,^13^ we found that interactions with HCAs are no more likely to be classified as negative than interactions with other staff types. A complex pattern is evident when exploring the interaction between staffing levels by RNs and HCAs. While higher levels of assistant staffing were associated with fewer negative interactions when RN staffing was moderate or high, when RN staffing was low, higher HCA staffing tended to be associated with increased levels of negative interactions, an important finding in an era in which cost efficiencies and RN supply difficulties are driving the deployment of a more dilute skill mix.^6–8^ Conversely, there was some indication that the effect of increased RN staffing is attenuated when HCA staffing is low.

Our findings may give an indication of the consequences of using assistant staff to compensate for an RN shortfall. They challenge the substitution of RNs with HCAs because they indicate that quality of interactions, and perhaps quality of care more generally, is not improved by adding HCAs to the workforce when there are insufficient RNs. The patterns observed may arise from the influence that supervising RNs have on the practice of HCAs, with inadequate supervisory capacity leading to adverse outcomes. Lack of supervisory capacity and unwarranted substitution has been hypothesised as a mechanism that might explain an association between higher HCA staffing and increased mortality,^37^ but more research is merited to better understand the mechanisms that explain how the staffing levels of RNs affect HCA practice and vice versa. These findings point to distinct and important contributions from RNs and from HCAs which cannot be understood through a single measure that combines both, such as the NHS Care Hours per patient day measure,^38^ because the effects of one staff group are contingent on the other.

There is ongoing international debate about whether setting minimum staffing levels across hospitals will improve quality of care and this policy has been resisted due to its lack of flexibility and potential to reduce innovation in workforce planning.^10 11 39^ Despite this, our results clearly indicate that when nurse staffing on adult hospital wards is above the threshold of eight patients per RN, identified as a ‘warning’ level by NICE,^9^ the likelihood of having a negative interaction is substantially increased. When this threshold is passed adding additional unregistered staff does not seem to mitigate the problem, rather it makes it worse. However, while we confirm worse outcomes when registered nurses care for eight or more patients, our measure of staffing is coarse with the top end of our reference category (eight patients per nurse) representing a 33% increase on the bottom (six patients per nurse). A larger sample may enable finer-grained conclusions.

While we did not find an association between staffing levels and amount of time patients spent interacting with staff totalled across all their interactions, we did find that increasing numbers of patients had a decreased rate of interactions with HCAs and increased rate with RNs. These findings suggest that high patient numbers affect the working patterns of RNs, resulting in more interactions of shorter duration. It may be that when patient numbers are high relative to numbers of staff, RNs place a higher priority on interacting with patients (as opposed to non-patient facing work) but that, in spite of this response, the resulting quality of interactions is reduced.

### Strengths and limitations

Ward-based staffing data and interaction data in real time overcomes the weaknesses of much previous survey-based research in the nursing workforce field which has been based on hospital-level averages.^6 7 40^ Our study provided an opportunity to study the actual staffing levels encountered by patients on wards. Data on staffing and care were prospectively gathered, rather than relying on secondary data or the recall of staff members. While it could be argued that data from independent observations of care may not be a suitable proxy for actual patient experience, there is a growing body of research supporting the use of QuIS for this purpose.^41 42^ Our success in including patients with cognitive impairment at a rate consistent with hospital inpatient populations in similar settings in other UK hospitals also overcomes the potential under-representation of patient groups when self-report methods are used to gauge patient experiences.^21 30 43 44^

Data gathered on ward and patient characteristics suggest that the six wards in this study are typical of similar NHS general acute care wards,^45^ but further study is merited in a wider range of inpatient settings, including internationally where nursing roles, patient mix and complexity may vary. The nature of our study has enabled a clearer link to be made between nurse staffing levels and outcomes than in previous observational research using surveys at hospital level, since it incorporated concurrent observation of patient care and staffing levels. Nevertheless, this remains an observational study and a causal inference cannot be directly made. The mechanism by which low staffing might generate an increased chance of negative interactions requires further exploration.

## Conclusion

Low RN staffing levels are associated with poorer quality interactions between patients and staff. In addition to the well-known patient safety risks, these findings indicate a wider negative effect from low staffing, with adverse consequences for patient experiences and quality of care more generally. We found some evidence for the benefit of increased HCA staffing. However, despite a number of current drivers that promote the substitution of RNs with less qualified staff, such substitutions may not be effective because adding HCAs when RN staffing levels were low was not associated with improvements. In the face of staff shortages, policies and practices that focus on improving the supply and retention of RNs, rather than substitution, may be less detrimental to quality of care. Nurse staff metrics, which treat RN and HCA hours as equivalent, can provide a significantly misleading picture of the ability of the nursing workforce to deliver a high quality of care.
